# A novel bocavirus in canine liver

**DOI:** 10.1186/1743-422X-10-54

**Published:** 2013-02-13

**Authors:** Linlin Li, Patricia A Pesavento, Christian M Leutenegger, Marko Estrada, Lark L Coffey, Samia N Naccache, Erik Samayoa, Charles Chiu, Jianming Qiu, Chunlin Wang, Xutao Deng, Eric Delwart

**Affiliations:** 1 Blood Systems Research Institute, San Francisco, CA, USA; 2 Department of Laboratory Medicine, University of California, San Francisco, CA, USA; 3 Department of Pathology, Microbiology and Immunology, School of Veterinary Medicine, University of California, Davis, CA, USA; 4 IDEXX Reference Laboratories, CA, USA; 5 UCSF-Abbott Viral Diagnostics and Discovery Center, CA, USA; 6 Department of Microbiology, University of Kansas, KS, USA; 7 Stanford Genome Technology Center, Stanford, CA, USA

**Keywords:** Canine bocavirus 3, Episome, Coinfection

## Abstract

**Background:**

Bocaviruses are classified as a genus within the *Parvoviridae* family of single-stranded DNA viruses and are pathogenic in some mammalian species. Two species have been previously reported in dogs, minute virus of canines (MVC), associated with neonatal diseases and fertility disorders; and Canine bocavirus (CBoV), associated with respiratory disease.

**Findings:**

In this study using deep sequencing of enriched viral particles from the liver of a dog with severe hemorrhagic gastroenteritis, necrotizing vasculitis, granulomatous lymphadenitis and anuric renal failure, we identified and characterized a novel bocavirus we named Canine bocavirus 3 (CnBoV3). The three major ORFs of CnBoV3 (NS1, NP1 and VP1) shared less than 60% aa identity with those of other bocaviruses qualifying it as a novel species based on ICTV criteria. Inverse PCR showed the presence of concatemerized or circular forms of the genome in liver.

**Conclusions:**

We genetically characterized a bocavirus in a dog liver that is highly distinct from prior canine bocaviruses found in respiratory and fecal samples. Its role in this animal’s complex disease remains to be determined.

## Background

Parvoviruses consist of small non-enveloped, autonomously replicating, single-stranded DNA viruses with genome length between 4.5 - 5.5 kb [1]. Bocavirus, a genus of the family *Parvoviridae,* is characterized by the presence of a third major ORF named NP1*.* Bocaviruses are known to infect multiple mammalian species including humans [2], cows [3], pigs [4-6], gorillas [7], chimpanzees [8], California sea lions [9], dogs [10-13], cats [11], bats [14], and pine martens [15]. Bocavirus infections can cause respiratory and gastrointestinal symptoms in young animals and humans, but are also often subclinical in adults [2,16]. While many bocaviruses were initially identified in feces or respiratory secretion they can also be found in blood [2,16].

Minute virus of canines (MVC) was the first known bocavirus infecting dogs. MVC was isolated in 1967 in the feces of a clinical healthy dog, and later recognized as causing neonatal diseases and fertility disorders in dogs [16]. The second species of dog bocavirus (Canine bocavirus, CBoV) was identified in 2011 in the respiratory samples from diseased and healthy dogs [10]. One genotype of CBoV was associated with respiratory disease as it showed higher prevalence in diseased animals than healthy controls [10]. Variants of this CBoV were also detected in fecal, nasal, urine and blood samples collected from dogs in Hong Kong [11].

In this study, an infectious etiology was suspected for a dog with severe hemorrhagic gastroenteritis, necrotizing vasculitis, granulomatous lymphadenitis and anuric renal failure. The clinical and post-mortem workups for infectious causes in this case included negative test results for Canine Parvovirus 2, Canine Enteric Coronavirus, Canine distemper virus, Salmonella, Campylobacter, Clostridium perfringens enterotoxin A gene, Cryptosporidium, and Giardia. Special stains of histologic specimens revealed no detectable bacteria or other known infectious agents. Using deep sequencing, we characterized viral sequences present in the dog’s liver revealing a third species of canine bocavirus described here.

## Results and discussion

A sequence of 300 bases showing sequence similarity to bocaviruses (BLASTx E < 10^-3^) was initially identified by 454 pyrosequencing. The sequence was extended by degenerate PCR targeting conserved bocavirus regions, yielding a ~2.5 kb partial genome sequence. The tissue nucleic acids were further analyzed using the MiSeq Illumina platform generating 16 contigs composed of 133 reads with similarities to bocaviruses (BLASTx E < 10^-3^), which allowed the amplification of all three ORFs. The virus was highly divergent from the other two known canine bocaviruses, MVC and CBoV, and was provisionally named Canine bocavirus 3 (CnBoV3) [10,11,13].

**Figure 1:**
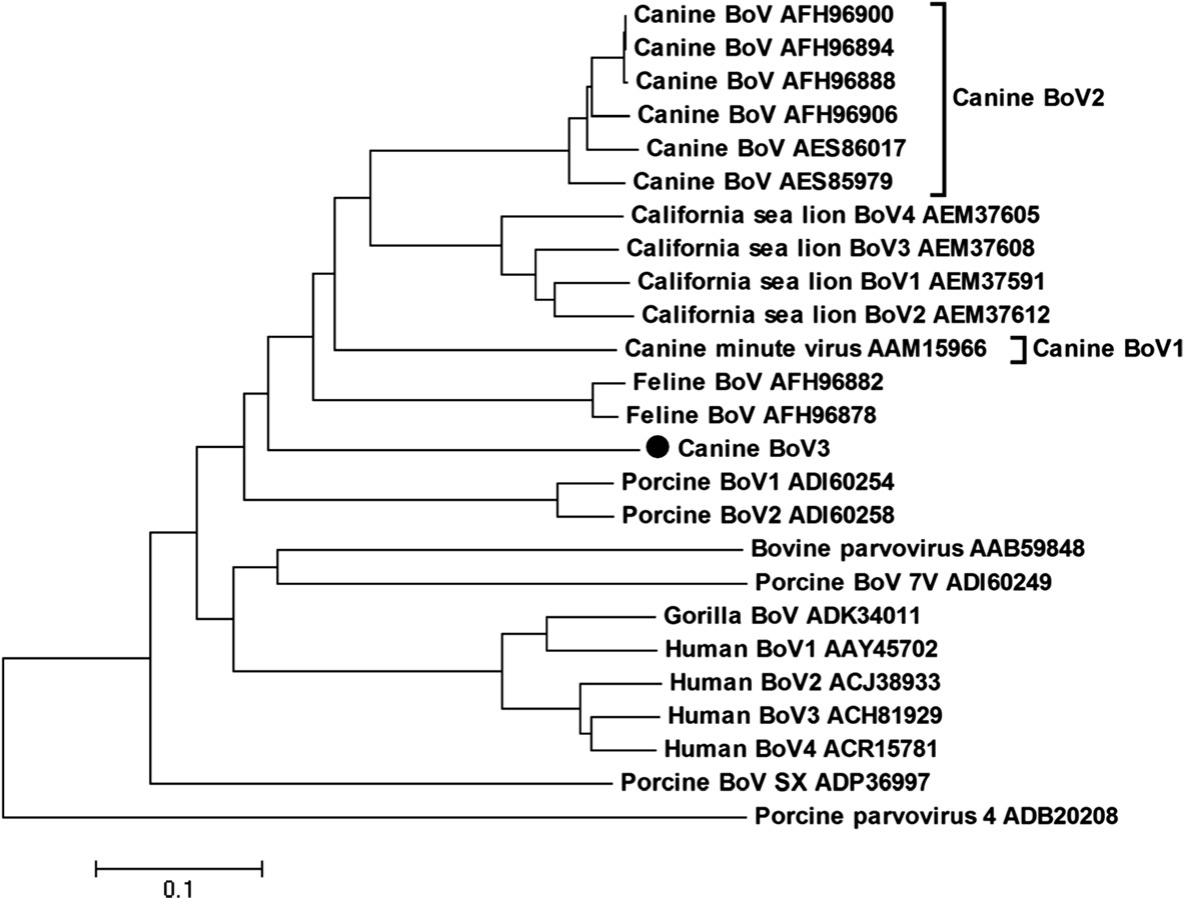
Phylogenetic tree based on aligned amino acid sequences of full-length VP proteins of representative bocavirus species.

The nonstructural (NS1) protein encoded by ORF1 was 778 aa long, and contained motifs associated with rolling circle replication, helicase and ATPase. The NP1 protein encoded by the middle ORF3 was 194 aa long. The ORF2 encoded capsid proteins VP1 (689 aa) and proteolytically processed VP2 (560 aa). CnBoV3 shared 51%, 57%, 56% aa similarity with the NS1, NP1 and VP1 region of CBoV, respectively, and 49%, 52%, 57% aa similarity with the NS1, NP1 and VP1 regions of MVC. Phylogenetic analysis of the entire VP1 was performed to determine the relationship between CnBoV3 and other bocaviruses. CnBoV3 was phylogenetically distinct from the known dog bocaviruses and only loosely related to California sea lion bocaviruses, CBoV, feline bocavirus and MVC (Figure 1). Phylogenetic trees constructed by NS1 and NP1 region yielded similar topology (data not shown).

Bocaviruses are believed to replicate through the parvovirus rolling hairpin model, which generate replication intermediates of concatemers with head-to-head or tail-to-tail structure [17]. Recent experimental evidence showed the presence of head-to-tail concatemers or circularized genomes of human bocavirus (HBoV) 1&3 [18,19] and porcine bocaviruses [20], indicating that some bocaviruses may use a rolling - circle replication model.

**Figure 2:**
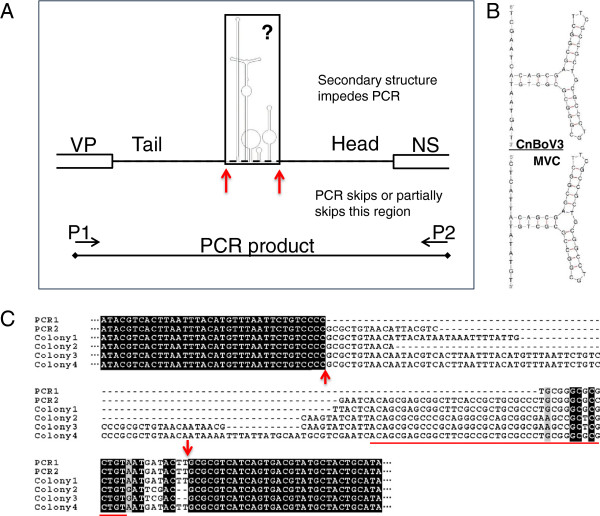
**A) Inverse PCR was performed to obtain the NTR sequence between the VP termination and the NS initiation codon of episomal forms of CnBoV3.** Primers (P1 and P2) were situated at VP and NS region respectively. Secondary structures (symbolic in the box) may have impeded the PCR resulting in several products of different length. **B)** Highly identical ‘rabbit ear’ structure of CnBoV3 and MVC. **C)** Alignments of NTR sequences obtained by sequencing different inverse PCR products directly (PCR1&2) and subcloning products (colony 1–4). Sequences outside the two arrows were identical. Underlined sequence shows location of “rabbit ear” sequence.

Using inverse PCR with primers directed outward from the 5’ and 3’ extremities of the partial genome sequence we were able to amplify head-to-tail sequence of the CnBoV3 non-translated regions (NTR) between the VP termination and the NS1 initiation codons (Figure 2A). The generation of specific PCR products indicated the presence of concatemerized or circular forms of the genome in the liver [18].

To confirm these results, multiple repeats of the inverse PCR were generated and directly sequenced as well as subcloned into a plasmid vector. PCR amplicons and plasmid inserts were Sanger sequenced using protocol for GC-rich/hairpin sequences. The resulting sequences were aligned and the length of the region between the VP stop and the NS start codon calculated. This region varied in length from 392 to 506 nt (Figure 2C). Mfold analyses of the longest NTR sequence (506 nt) showed that the long palindromic hairpin terminal repeats (e.g. approximately 150 nt at both ends in MVC or bovine parvovirus, BPV) were missing, but a typical “rabbit ear” structure was detected that was nearly identical to a structure in the MVC 5'NTR sequence (Figure 2B). These results suggested that inverse PCR may have been impeded by strong secondary DNA structures (Figure 2A &C, between two red arrows). None of the currently reported NTR regions of bocavirus obtained by inverse PCR contained both complete inverted terminal repeats seen in MVC or BPV [18-20].

## Conclusions

In this study we genetically characterize a third species of canine bocavirus (CnBoV3), highly divergent from MVC [21] and CBoV [10,11], in the liver of a dog with severe disease. The presence of three distinct canine bocaviruses, phylogenetically interspersed with viruses from different mammalian hosts, may reflect their origins from different cross-species transmissions. The detection of CnBoV3 in liver tissue indicated that the virus had likely breached the mucosal barrier of the typical sites of bocavirus replication in the respiratory or enteric tracts. The detection of episomal forms by PCR indicated that replication may be occurring in hepatocytes or other liver cell types and that viremia was also likely present although the lack of available blood sample prevented direct testing. We also detected in that animal co-infection with a canine circovirus. Circovirus infection can lead to lymphocytic depletion and immunosuppression in the host [22]. Whether CnBoV3, canine circovirus, or their combination were involved in this dog’s severe symptoms requires further investigation.

## Materials and methods

A dog suffering from vomiting and hemorrhagic gastroenteritis was euthanized after a rapid disease course lasting seven days. Necropsy showed the presence of necrotizing vasculitis, granulomatous lymphadenitis and anuric renal failure. Clinical and post-mortem tests for multiple infectious agents of enteric disease were all negative. Liver Tissue was stored at −80°C until further processing. Tissue samples (~25 mg) were homogenized, filtered, and nuclease treated as previously described to enrich for nucleic acids within viral particles [23]. Nucleic acids were then extracted using the QIAamp viral RNA Mini kit (Qiagen), randomly amplified using random RT-PCR with randomized 3’ primers and nucleic acid libraries prepared as previously described [24] for sequencing using the Genome Sequencer FLX Instrument (454 Life Science, Roche). The pyrosequencing reads were sorted, trimmed, assembled and compared to the GenBank non-redundant databases as previously described [9]. Potential viral sequences were identified with translated protein sequence similarity matches (BLASTx to GenBank's NR database with E-value < 10^-3^) to known viral sequences. The presence of virus protein sequences detected by 454 pyrosequencing was confirmed by PCR and Sanger sequencing. Genome walking and degenerate PCR were used to amplify the viral genome. Enriched viral nucleic acids from the infected tissue was also handled using the ScriptSeq RNA-Seq library preparation kit (Epicentre) and sequenced by MiSeq system (illumina). The resulting near complete genome of CnBoV3 was deposited in GenBank with accession no. KC580640. Phylogenetic analyses based on aligned amino acid sequences from full-length VP proteins were generated by the neighbor joining method in MEGA [25], using amino acid p-distances, with 1,000 bootstrap replicates.

## Competing interests

The authors declared that they have no competing interests.

## Authors’ contributions

LL generated the data, interpreted the data and drafted the manuscript. PP selected and collected the animal sample and helped interpret results. CL and ME performed real-time PCR. LC, SN, ES and CC provided help with Illumina sequencing. CW and XD provided bioinformatics analyses of 454 and Illumina data. JQ assisted in the NTR analysis. ED directed the research and revised the draft. All authors read and approved the final manuscript.

## References

[B1] Tattersall P Bergoin M Bloom M Brown K Linden R Tijssen P Fauquet C, Mayo M, Maniloff J, Desselberger U, Ball L Virus Taxonomy: Eighth Report of the International Committee on Taxonomy of Viruses 2005 San Diego: Academic 353 369

[B2] Jartti T Hedman K Jartti L Ruuskanen O Allander T Soderlund-Venermo M Human bocavirus-the first 5 years Rev Med Virol 2012 22 46 64 10.1002/rmv.720 22038931

[B3] Chen KC Shull BC Moses EA Lederman M Stout ER Bates RC Complete nucleotide sequence and genome organization of bovine parvovirus J Virol 1986 60 1085 1097 3783814 10.1128/jvi.60.3.1085-1097.1986 PMC253350

[B4] Cheng WX Li JS Huang CP Yao DP Liu N Cui SX Jin Y Duan ZJ Identification and nearly full-length genome characterization of novel porcine bocaviruses PLoS One 2010 5 e13583 10.1371/journal.pone.0013583 21049037 PMC2963602

[B5] Shan T Lan D Li L Wang C Cui L Zhang W Hua X Zhu C Zhao W Delwart E Genomic characterization and high prevalence of bocaviruses in swine PLoS One 2011 6 e17292 10.1371/journal.pone.0017292 21525999 PMC3078135

[B6] Shan T Li L Simmonds P Wang C Moeser A Delwart E The fecal virome of pigs on a high-density farm J Virol 2011 85 11697 11708 10.1128/JVI.05217-11 21900163 PMC3209269

[B7] Kapoor A Mehta N Esper F Poljsak-Prijatelj M Quan PL Qaisar N Delwart E Lipkin WI Identification and characterization of a new bocavirus species in gorillas PLoS One 2010 5 e11948 10.1371/journal.pone.0011948 20668709 PMC2909267

[B8] Sharp CP LeBreton M Kantola K Nana A Diffo Jle D Djoko CF Tamoufe U Kiyang JA Babila TG Ngole EM Widespread infection with homologues of human parvoviruses B19, PARV4, and human bocavirus of chimpanzees and gorillas in the wild J Virol 2010 84 10289 10296 10.1128/JVI.01304-10 20668071 PMC2937811

[B9] Li L Shan T Wang C Cote C Kolman J Onions D Gulland FM Delwart E The fecal viral flora of California sea lions J Virol 2011 85 9909 9917 10.1128/JVI.05026-11 21795334 PMC3196430

[B10] Kapoor A Mehta N Dubovi EJ Simmonds P Govindasamy L Medina JL Street C Shields S Lipkin WI Characterization of novel canine bocaviruses and their association with respiratory disease J Gen Virol 2012 93 341 346 10.1099/vir.0.036624-0 22031527 PMC3352345

[B11] Lau SK Woo PC Yeung HC Teng JL Wu Y Bai R Fan RY Chan KH Yuen KY Identification and characterization of bocaviruses in cats and dogs reveals a novel feline bocavirus and a novel genetic group of canine bocavirus J Gen Virol 2012 93 1573 1582 10.1099/vir.0.042531-0 22495233

[B12] Ohshima T Kawakami K Abe T Mochizuki M A minute virus of canines (MVC: canine bocavirus) isolated from an elderly dog with severe gastroenteritis, and phylogenetic analysis of MVC strains Vet Microbiol 2010 145 334 338 10.1016/j.vetmic.2010.03.033 20427134 PMC7117362

[B13] Schwartz D Green B Carmichael LE Parrish CR The canine minute virus (minute virus of canines) is a distinct parvovirus that is most similar to bovine parvovirus Virology 2002 302 219 223 10.1006/viro.2002.1674 12441065

[B14] Wu Z Ren X Yang L Hu Y Yang J He G Zhang J Dong J Sun L Du J Virome analysis for identification of novel mammalian viruses in bat species from Chinese provinces J Virol 2012 86 10999 11012 10.1128/JVI.01394-12 22855479 PMC3457178

[B15] van den Brand JM van Leeuwen M Schapendonk CM Simon JH Haagmans BL Osterhaus AD Smits SL Metagenomic analysis of the viral flora of pine marten and European badger feces J Virol 2012 86 2360 2365 10.1128/JVI.06373-11 22171250 PMC3302375

[B16] Manteufel J Truyen U Animal bocaviruses: a brief review Intervirology 2008 51 328 334 10.1159/000173734 19023216

[B17] Schildgen O Qiu J Soderlund-Venermo M Genomic features of the human bocaviruses Future Virol 2012 7 31 39 10.2217/fvl.11.136 22389649 PMC3291126

[B18] Kapoor A Hornig M Asokan A Williams B Henriquez JA Lipkin WI Bocavirus episome in infected human tissue contains non-identical termini PLoS One 2011 6 e21362 10.1371/journal.pone.0021362 21738642 PMC3125170

[B19] Lusebrink J Schildgen V Tillmann RL Wittleben F Bohmer A Muller A Schildgen O Detection of head-to-tail DNA sequences of human bocavirus in clinical samples PLoS One 2011 6 e19457 10.1371/journal.pone.0019457 21573237 PMC3087758

[B20] Yang WZ Huang CP Duan ZJ [Identification and characterization of porcine bocavirus episomes] Bing Du Xue Bao 2012 28 418 423 22978168

[B21] Binn LN Lazar EC Eddy GA Kajima M Recovery and characterization of a minute virus of canines Infect Immun 1970 1 503 508 16557766 10.1128/iai.1.5.503-508.1970 PMC415932

[B22] Segales J Porcine circovirus type 2 (PCV2) infections: clinical signs, pathology and laboratory diagnosis Virus Res 2012 164 10 19 10.1016/j.virusres.2011.10.007 22056845

[B23] Victoria JG Kapoor A Dupuis K Schnurr DP Delwart EL Rapid identification of known and new RNA viruses from animal tissues PLoS Pathog 2008 4 e1000163 10.1371/journal.ppat.1000163 18818738 PMC2533695

[B24] Victoria JG Kapoor A Li L Blinkova O Slikas B Wang C Naeem A Zaidi S Delwart E Metagenomic analyses of viruses in stool samples from children with acute flaccid paralysis J Virol 2009 83 4642 4651 10.1128/JVI.02301-08 19211756 PMC2668503

[B25] Kumar S Nei M Dudley J Tamura K MEGA: a biologist-centric software for evolutionary analysis of DNA and protein sequences Brief Bioinform 2008 9 299 306 10.1093/bib/bbn017 18417537 PMC2562624

